# Non-viral gene delivery systems for tissue repair and regeneration

**DOI:** 10.1186/s12967-018-1402-1

**Published:** 2018-02-15

**Authors:** Pan Wu, Haojiao Chen, Ronghua Jin, Tingting Weng, Jon Kee Ho, Chuangang You, Liping Zhang, Xingang Wang, Chunmao Han

**Affiliations:** Department of Burns & Wound Care Center, Second Affiliated Hospital of Medical College, Zhejiang University, Hangzhou, 310009 China

**Keywords:** Non-viral vector, Tissue engineering, Gene therapy

## Abstract

Critical tissue defects frequently result from trauma, burns, chronic wounds and/or surgery. The ideal treatment for such tissue loss is autografting, but donor sites are often limited. Tissue engineering (TE) is an inspiring alternative for tissue repair and regeneration (TRR). One of the current state-of-the-art methods for TRR is gene therapy. Non-viral gene delivery systems (nVGDS) have great potential for TE and have several advantages over viral delivery including lower immunogenicity and toxicity, better cell specificity, better modifiability, and higher productivity. However, there is no ideal nVGDS for TRR, hence, there is widespread research to improve their properties. This review introduces the basic principles and key aspects of commonly-used nVGDSs. We focus on recent advances in their applications, current challenges, and future directions.

## Background

Critical tissue defects are clinically common because of trauma or pathology such as extensive burns and non-union bone fractures [1, 2]. The standard treatment for such tissue loss is autografting [3]. However, the supply of donor tissues is often limited and the capability of self-repair following damage is insufficient or delayed [4]. Tissue engineering (TE) is an inspiring alternative for tissue repair and regeneration (TRR) [5]. Cells, biomolecules, and biomaterials have been widely used to induce in situ wound healing and tissue regeneration, or to produce in vitro TE constructs [6]. The delivery of proteins such as growth factors [7] generally exhibit shortcomings such as short half-life times, large dosages, and high costs of the delivered molecules [8]. To avoid these drawbacks and further improve TRR, therapeutic nucleic acids (NAs), e.g. plasmid deoxyribonucleic acids (pDNAs), small interfering ribonucleic acids (siRNA) and microRNAs (miRNA), provide better promotion of high-quality TRR [9].

The advantages of nucleic acid (NA)-based TRR include: (1) more sustained expression of the encoded genes, or prolonged up-regulation or down-regulation of the targeted genes exerted by RNA interference (RNAi); (2) exemption from immunologic reaction; (3) improved cost efficiency. A variety of methods can be used to deliver NAs into target cells, e.g. physical techniques, viral vectors (VVs) and chemical or biochemical vectors [10]. The physical methods, e.g. gene gun, electric perforation and ultrasonic are usually realised within a small number of cells at one time, and the manipulated cells often show low activity because of physical damage, resulting in poor TRR. Viral vectors such as adenovirus, lentivirus, and retrovirus, however, can transfect cells in large quantities, achieve high transfection rates and generate life-long expression of the transgenes or regulation of the host genome. But insertion mutation and other adverse effects have been observed during the post-transfection periods after viral gene delivery [11], resulting in the uncertainty and controversy about the application of VV for TRR.

Chemical or biochemical vectors, also known as non-viral vectors (nVVs), are primarily composed of two groups of vectors, namely organic and inorganic vectors. The former consists of lipid-based vectors, natural and synthetic polymers and peptide-based vectors [12–14]. There are a variety of types of nVVs that are commonly used with Lipofectamine 2000 [15] and polyethylenimine (PEI) [16, 17] as the gold standard for sufficient transfection efficiency. Inorganic vectors mainly include calcium phosphates (CaPs) [18] and metal nanoparticles [19]. These nVVs have several advantages over VVs, such as lower immunogenicity and toxicity, better cell specificity, better modifiability and enhanced productivity [20]. They are a better alternative to deliver genes responsible for the repair and regeneration of damaged tissues. However, the transfection efficiency of an nVV is often lower, restricting its application compared to a VV [21] and varies depending on the type of vector and target cell.

Scaffolds or matrices in TE are primarily designed to preserve tissue volume and provide a sequential transition during which the regenerated tissue assumes function as the scaffold degrades. Porous scaffolds are good reservoirs for the release of biomolecules and, furthermore, provide structural support for cells to adhere and proliferate during migration toward the center of the wound [22]. Scaffolds/matrices loaded with vector-gene complexes (VGCs) or naked NAs are termed gene-activated matrices (GAMs) [23]. GAMs with VGCs seem to be more suitable for gene delivery because of their better protection of NAs, and they excel at local and controlled delivery of VGCs in the specific region of the damaged tissue [24]. This avoids off-target effects and increases the efficiency of gene transfer.

A typical model of the state-of-the-art non-viral gene delivery system (nVGDS) for TRR is composed of three major elements: scaffold or matrices, cells including stem cells, and non-viral vector/gene complexes (nVGCs) [25]. However, there is no perfect engineered tissue that has the identical properties of normal tissues. Optimisation of existing TE constructs or development of novel TE products, especially those involving nVGDS, is a major trend in the field. Herein, we introduce the basic principles and key aspects of commonly used nVGDSs, followed by descriptions of recent advances in their applications, current challenges, and future directions.

## Mechanism of non-viral gene delivery

**Fig. 1:**
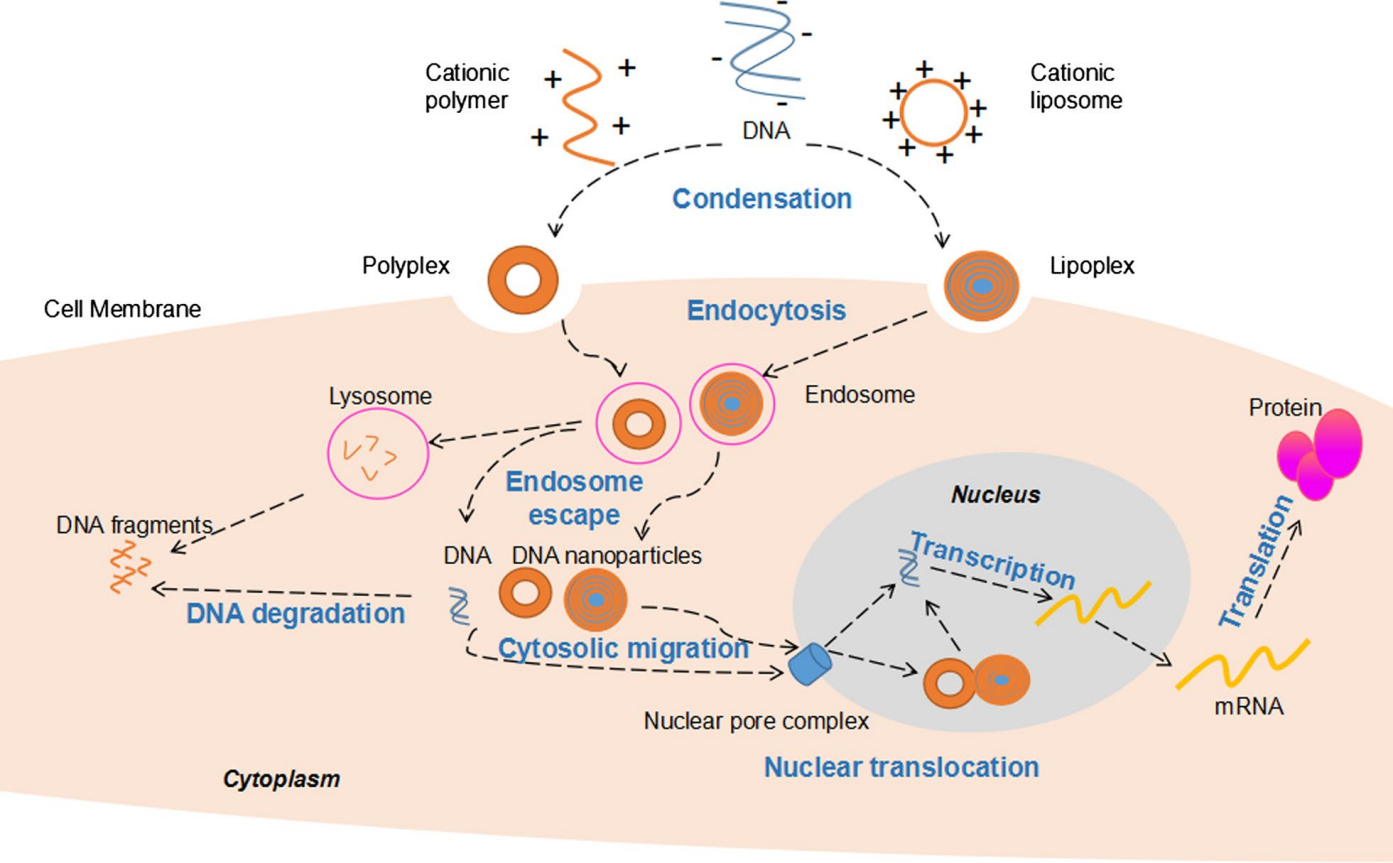
Basic mechanism of non-viral gene delivery via polyplex and lipoplex. DNA is condensed via interaction with a cationic polymer or encapsulated in a cationic liposome to form a polyplex or lipoplex and pass through the cell membrane via endocytosis. Once endosome escape occurs, the complex is released into the cytosol, the released DNA will be transported to the perinuclear region via microtubule system. Nuclear translocation of the exogenous DNA can be achieved by passage through nuclear pore complex in non-dividing cells. After right transcription and translation, target proteins are produced to exert biological effects

Gene delivery is the transfer of exogenous NA from the extra-cellular environment to intracellular compartments, i.e. the nucleus for pDNA or cytoplasm for siRNA or miRNA. The detailed mechanism of non-viral gene delivery (nVGD) is still not clear. However, it can be generally divided into five stages through which the cargo overcomes several biological barriers, i.e. the extra-cellular environment, cell membrane, endolysomal system, nuclear envelope, and transcription/translation interruptions [26]. We use the typical scaffold/nVGC-pDNA/cell system to illustrate the multi-stage process of nVGD (Fig. 1).

### Extra-cellular gene delivery

pDNA condensed and incorporated with nVV to form nanoparticles is an nVGC that can provide better protection against DNAase [27]. The nVGC is later loaded onto a TE scaffold, consisting of biomaterials, by soaking or via a biochemical reaction generating a GAM [28, 29]. When the GAM is applied to a tissue defect, the nVGC can be released into the local extra-cellular environment or the surrounding cells that gradually adhere to and migrate along the scaffold to meet the nVGC [28].

### Cellular uptake

Once contact between the nVGC and the target cells is achieved, interactions are triggered on the cell surface. The nVGC can then pass through the cell membrane by cellular uptake via endocytic or non-endocytic pathways [30]. Non-endocytic pathways include invasive and non-invasive systems [31], while endocytic pathways are more common in nVGD. These include phagocytosis, clathrin-mediated endocytosis (CME), caveolae-mediated endocytosis (CvME), and macropinocytosis [32]. The initial signal of the endocytic pathway may be the ligand–receptor reaction [33]. The nVGC interacts with the cell membrane, becomes partially encapsulated, dissociates to form intracellular vesicles, and finally integrates into endosomes [34].

### Endolysosomal escape

After entering the cytoplasm in the form of an intracellular vesicle, the encapsulated CVGC should release from the endosomes into the cytoplasm, otherwise the nVGC will be destroyed by the acidic environment of the endosomes [35]. This releasing process is called endosomal escape [36]. The common synthetic cationic polymer PEI has a high buffering capacity between physiological and endosomal pH, which leads to endosomal escape [37]. Specifically, PEI loaded with pDNA can cause an increase in osmotic pressure within endosomes, leading to the disruption of the endosomal membrane, thereby releasing the nVGC into the cytoplasm [38].

### Nuclear translocation

The released nanosized nVGC may trigger autophagy before achieving complete nuclear entrance [35]. The disassociation of pDNA from nVGC occurs in some cases, and pDNA released from the nVGC may also be degraded by DNase in the cytosol. Thus, it is crucial for successful nuclear translocation of pDNA to ensure final correct expression [26]. Two possible pathways toward nuclear transportation are suggested in the literature, i.e. microtubule-dependent and -independent pathways [39].

Nuclear entry of pDNA, either in the form of nVGC or pDNA alone, can be achieved by cell-dividing-dependent or cell-dividing-independent pathways [40]. In the cell-dividing-dependent pathway, nVGCs, especially large ones, e.g. polyplexes (complexes composed of pDNA and polymers), enter the nucleus when the nuclear envelope breaks down during the G2/M transition of the dividing cell [41]. In the latter pathway, the nVGC mainly enters the nucleus through the nuclear pore complex (NPC) [42]. Nuclear localisation signals, e.g. the classical nuclear location sequence (NLS) from the SV40 Large T-antigen (PKKKRKV) and the bipartite NLS (typically KKKX5-20RK), are borne in the cargo protein, which can carry the intracellular pDNA toward NPC via interaction with importin. And when the pDNA-protein complex traffics to the NPC, the importin will interact with the NPC and facilitate the translocation across the pore [43].

### Transcription and translation

Transcription is the process of biosynthesis of RNA that is carried out on the DNA template in the nucleus. It is initiated by the interaction of the transcription factor in the nucleus and the promoter in the delivered pDNA [44]. Then, the mRNA is transported out to the cytosol to be translated into the corresponding protein. To some extent, high transfection rates can be reached more easily, but the amount of the eventually expressed function molecules remains low or even undetectable [45] due to insufficient transcription and/or translation. Research concerning this process in the field of nVGDS remains inadequate.

### Factors related to nVGD

Factors that affect nVGD can be classified into three groups with respect to the different components of nVGD: gene/vector complexes, cells, and the interactions between them. Commonly discussed characteristics of gene/vector complexes include particle size, charge density, N/P ratio, and molecular weight [34, 46, 47]. Primary cells and stem cells are considered difficult to be successfully transfect. Recent investigations have studied the three-dimensional (3D) structure and functional groups of nVVs [48]. Tremendous efforts have been made to facilitate transfection of these hard-to-transfect cells for TRR [49]. Improved interaction between the nVGDS components may significantly exert impacts on the efficiency and safety of non-viral gene vectors [50]. Additionally, one factor may play a role in several stages during the gene delivery process [48, 51].

Several molecules are responsible for enhanced cellular uptake rates. Cell-penetrating proteins (CPPs) are of particular interest among the cellular affinity regulatory factors. They contain positively charged amino acid residues, such as arginine and lysine, capable of translocating various macromolecules across the plasma membrane and targeting the cell nucleus with no obvious toxicity. They are widely used to enhance the transfection efficiency [52]. Glycosaminoglycans (GAGs), previously considered as being important substances participating in cell surface binding [53], may alter the intracellular pathway and induce substantial pDNA elimination [54].

Different kinds of vectors participate in cellular uptake via different pathways. Most cationic polymers enter cells via the CME pathway, while compensatory endocytic mechanisms also exist. It has been demonstrated that the cationic polymer PEI and dendrimer polyamidoamine (PAMAM) are internalised via both CME and CvME pathways. The inhibition of one endocytic pathway may lead to an overall increase in uptake via unaffected pathways [55].

Factors that can increase the buffering capacity of the nVV are favoured in the process. Gene expression enhancers are important in nVGDS to facilitate endosomal escape. However, they can also increase the cytosolic DNase level by endosomal eruption, which will destroy the delivered DNA [56].

During nuclear translocation, various molecules have been identified that facilitate completion of the process. The function of microtubules, importins, and NLSs are important [39]. In the cell-dividing-dependent pathway, nuclear entry of nanoparticles or naked DNA is closely related to mitosis, which is also the main mechanism of a viral vector entering the nucleus [57]. However, the risk of mutation insertion exists. In the cell-dividing-independent pathway, several factors are involved, including the DNA nuclear targeting sequence (DTS), transcription factors such as AP1, AP2, nuclear factor (NF)-κB, Oct1, TEF-1, NLS, amphiphilic block copolymers, and importin [40]. The DNA-bound NLSs with binding sites for the transcription factor NF-κB and NLS enhancers can be recognised by importin and translocate across the NPC. Some macromolecules can guide their incorporated CVGC to nuclei via interaction of their cytoplasmic binding proteins and their receptors near the NPC, e.g. Vitamin A [58]. Tanaka et al. further demonstrated that a series of newly developed compounds containing vitamin A, which were referred to as a vitamin A-scaffold SS-cleavable proton-activated lipid-like materials (SSPalms), could facilitate the nuclear import of pDNA in the form of lipid nanoparticles (LNPs) [59].

Factors that may have an important role in transcription and translation include transcription factors, NLS, and others [44]. However, the detailed mechanisms remain unclear.

## Non-viral gene delivery system design

Based on the advanced understanding of nVGDSs, several factors should be considered in their design to increase transfection efficiency and decrease cytotoxicity. These include DNA condensation, complex stability, membrane activity, cellular uptake, endosome buffering capacity, vector degradability, and targeting property (Table 1). Additionally, combinations of different kinds of materials or transfection enhancers can significantly increase the efficiency and reliability, and minimise side effects [60].

**Table 1:** Modifications for novel non viral gene delivery vectors

Function	Target stage	Description	Examples	References
Increase transfection efficiency	DNA condensation	Hydrophobic moieties	Polyethylene glycol (PEG)	[27, 48, 61, 64]
	Complex protection	Hydrophobic side chains	PEG	[27, 48, 61, 63]
		Imidazole groups		[76]
	Cellular uptake	Cell penetrating peptides (CPPs)	Arginine	[68–71]
		CPP like proteins	Glucosamine residues	[72–74]
			Syndecans	[75]
		Long hydrophobic chains	PEG	[48]
	Endosolysomal escape	Increase buffering capacity	Poly(l-histidine)	[185]
			Imidazole groups	[76]
			Glycosides	[78]
			Xylitol	[77]
			Cyclodextrins	[85]
			Glycerol	[36]
			Hydroxyl groups	[80]
	Nuclear translocation		Nuclear location sequence	[81]
	Transcription and translation		Transcriptional factors	[45]
	Balancing buffering capacity and cytotoxicity	Hydrophobic side chains	PEG	[62, 65]
			Guanidine groups	[27]
Decrease adverse effects	Degradability	Decrease cytotoxicity	Imidazole groups	[76]
	Tissue/cell targeting property	Peptides	ATS-9R	[82]
			RGV	[83]
			Mannose	[52]
			Tet1	[84]
			Melittin	[85]
	Stimuli-responsive moieties	Biochemistry reaction	Disulfide bonds	[186]
			Nitrobenzene moiety	[29]
Other novel nVV		Inorganic nVV	Graphene	[87]
			Inorganic coating of calcium phosphate (CaP)	[88]
			Layered double hydroxide (LDH)	[89, 90]
			SiO_2_@LDH core–shell nanoparticles	[91]
		Combinatorial nVV	Cationic polymers and liposomes	[93]
			Nanoporous silicon-PEI nanoparticles	[86]
			Magnetic nanoparticles	[92]
			PAMAM conjugated gold nanoparticles (AuPAMAM)	[19]

### Improve transfection efficiency

The expression rate of a target protein is a key metric for transfection efficiency [9]. The approach for improving transfection efficiency varies according to the different stages targeted during transfection.

#### DNA condensation and complex stability

The first step in nVGD is condensation and protection of DNA during which the DNA should be nanosized for safe transport across the barriers. Hydrophobic moieties such as poly(ethylene glycol)s (PEGs) can improve condensation and complex stability. A PEG can increase the hydrophilicity of a substance, and hydrophobic side chains may provide a protective function [27, 61]. The cationic helical polypeptide-based conjugate poly(g-4-((2-(piperidin-1-yl)ethyl)aminomethyl)benzyl-l-glutamate, which displays excessive membrane activity leading to irreversible damage to cell membranes, can be modulated structurally by PEGylation to alter the complexation capacity with DNA, interaction with cellular/endosomal membranes and, ultimately, the transfection efficiency [48]. In another study, a fibre sheath of poly(dl-lactide)-PEG, which enhanced the structural integrity and maintained the biological activity of pDNA during the electrospinning process, was incorporated with varying amounts of PEG; it showed sustained release of pDNA polyplexes, and the effective release lifetime could be controlled to between 6 and 25 days. Furthermore, it was demonstrated that fibres loaded with pDNA polyplexes containing 10% PEG showed the best performance in terms of balancing transfection efficiency and cell viability [62]. Concerning lipid-based vectors, PEG can be used to form a protective coating on the surface of a vector, which is then likely to be opsonised and eliminated in the serum; and several PEGylated galactosylated cationic liposomes have been developed [63].

Short hydrophobic moieties not only improve the efficiency of transfection but also have a significant effect on the condensation process and the subsequent properties of the formed systems [64]. Reports indicate that long hydrophobic chains can improve the internalisation of the complexes through interaction with the cell membrane, however, they may also cause cytotoxicity [65]. One research group added charged groups, such as guanidine groups, to the side chain of cationic helical polymers, yielding elongated hydrophobic side chains and increased membrane activity, but higher toxicity [27]. Further experiments optimising the structure of the polymer were conducted by varying the amount of guanidine, and it was observed that an excess amount of guanidine diminished the transfection rate possibly via the mechanism of cell membrane penetration [27]. In a study of terpolymers incorporating different lengths of hydrophobic side chains, the best-performing hydrophobic terpolymers markedly enhanced the transfection activity relative to the polymers synthesised without alkylamine [66].

#### Membrane activity and cellular uptake

Membrane activity determines the cellular uptake rate of an nVV. The most commonly incorporated molecules in designing novel nVVs are cell-penetrating peptides (CPPs). These are a group of peptides that can enter cells by crossing the plasma membrane directly, or through uptake via the endocytotic pathway. They can be used as nucleic acid vectors or used as a modification method to enhance the membrane activity of non-viral gene vectors (nVGVs) [67]. Traditional CPPs are exemplified by HIV-TAT, Arg9, NLS, and Penetratin [68]. As an example, α-cyclodextrin (CD), a derivative of a natural cationic polymer, was modified with octa-arginine (CDR). This modification had excellent cell-penetrating ability and could be incorporated into a CDR/Az-I-Dex/DNA polyplex delivery system [69]. In another report, an arginine-terminated PAMAM nanoparticle-based nVGDS successfully reprogrammed fibroblasts to pluripotency [70]. Conjugated peptides with CPPs are also used in nVGD. A modified peptide termed RALA, in which the lysine residues were replaced with arginine, showed lower cytotoxicity via retaining its pH sensitivity, potentially by improving binding to the negatively charged outer leaflets of membranes and nucleic acids, and displayed better transfection efficiency [71].

The cell-penetrating helical polymer poly(γ-4-(((2-(piperidin-1-yl)ethyl)amino) methyl)benzyl-l-glutamate) (PVBLG-8) was also developed and optimised for better balance of the transfection efficiency and cytotoxicity by incorporating poly(γ-glucosamine methyl)benzyl-l-glutamate (PVBLG-7), a helical polypeptide bearing glucosamine residues, to form the PVBLG-8/PVBLG-7/DNA ternary complexes via self-assembly [72–74]. Similarly, CPP-like proteins can modify lipid-based delivery vectors, e.g. syndecans, which constitute a highly conserved family of transmembrane heparan sulphate proteoglycans. These serve as attachment sites for a great variety of cationic ligands including growth factors, cytokines, and even parasites, and can contribute to lipoplex-mediated gene delivery as cell-penetrating proteins [75].

#### Endosome buffering capacity

Increasing endosome buffering capacity is one of the most important modifications of nVVs. Numerous attempts have been made to add functional moieties to facilitate endosome escape. Poly(l-histidine), which has many imidazole groups with a pKa of about 6.0, can absorb protons and has a buffering capacity in the endosomal pH range (pH 5–6.5), leading to osmotic swelling and increased escape of pDNA. The large capacity of proton buffering at endosomal/lysosomal pH by a imidazole group-modified nVGDS provided a promising transfection efficiency of > 80% while reducing the cytotoxicity and enhancing the stability of the complexes [76].

Carbohydrates and their derivatives, such as glycosides, xylitol, and cyclodextrins, can modulate endosomal osmolysis or membrane permeability. One example is that of crosslinking xylitol diacrylate to low molecular weight PEI to form a polyxylitol-based gene carrier (XGC). A small amount of xylitol (3.9%) contributed 50% of the osmosis to XGC-inducing endosome eruption and improved endosomal escape [77]. SO1861, a natural glycoside, composed of a hydrophobic triterpene backbone and branched carbohydrate chains and a transfection enhancer, was integrated into a lipid-protamine-DNA (LPD) matrix, forming an SO1861-sensitised LPD (LPDS) that enhanced endosomal escape capacity and improved the transfection rate [78]. Cyclodextrins have both hydrophilic cavity exteriors and apolar cavity interiors, providing a micro-environment for encapsulation and solubilisation of hydrophobic “guest” molecules. They can be used to modify nVGDSs to increase membrane permeability through complexation with membrane phospholipids and cholesterols [79]. Hydroxyl groups, bearing many polysaccharides, affect delivery efficiency and serum tolerance of poly(glycoamidoamine)s [80].

Glycerol, which is an intermediate in carbohydrate and lipid metabolism, can be used as an addition to nVGDS to modify the buffering capacity and reduce cytotoxicity. Singh et al. developed two novel nVVs by crosslinking glycerol molecules with low molecular weight PEI, namely HG-PEI (45 mol% glycerol content) and LG-PEI (9 mol% glycerol content). Both vectors had similar DNA binding, DNA unpacking, and cellular uptake abilities but differed in buffering capacity. The cellular uptake and subsequent transfection efficiency of the LG-PEI was superior to that of the commercially available 25 kDa PEI, while HG-PEI demonstrated a lower transfection efficiency but higher cellular uptake rate [36].

#### Nuclear targeting

Nuclear location sequence (NLS) is a sequence that can enhance transfection efficiency via increasing nuclear uptake and nuclear translocation. NLSs have been widely used to modify nVGVs, e.g. the pH-sensitive core–shell system FA-PEG-CCTS/PAMAM/HMGB1/pDNA nanocomplexes (FPCPHDs) [60] and a histone H1-based recombinant fusion peptide with a nuclear localisation signal from human immunodeficiency virus, to enhance translocation of pDNA toward the cell nucleus [81].

### Targeting moieties

The targeting design of an nVV is a way to limit reaction in local tissue. Some peptides have the ability to demonstrate a target delivery. For example, ATS-9R, an adipocyte-targeting sequence, was combined with a short-hairpin RNA (shRNA) for silencing the fatty-acid-binding protein shFABP4. The ATS-9R/shFABP4 oligopeptide complex targeted mature adipocytes via binding to inhibitin and silencing of the targeted sequence peptide resulted in reduced lipidosis [82]. A 29-amino acid cell-binding peptide, RGV, conjugated to the redox-sensitive biodegradable dendrimer PAM-ABP, provided a low toxicity compound that increased transfection rates of both hMSC and hESC (about 60 and 50%, respectively) and retained expression of pluripotent stem cell markers [83]. Mannosylated CPP was bound to PEI to form the polymer Man-PEI1800-CPP, which was targeted at the mannose receptor on antigen-presenting cells (APC); its transfection rate was higher than 25KDa PEI but with lower toxicity, and in vivo experiments showed that this kind of nVGDS was mainly distributed in the epidermis and dermis [52]. The Tet1 peptide can bind to gangliosides highly expressed on GT1b neurons. When Tet1 was grafted to polypeptides in different amounts and constructed with oligopeptide and HPMA, it transfected neuron-like PC-12 cells with an increase in transfection rate and no cytotoxicity increase was found [84]. Grafting melittin, a 26 amino acid peptide, to HPMA-oligolysine formed a polymer that transfected HeLa cells and PC-12 cells. Because of its membrane lysis capacity, the melittin-grafted polymer had increased cytotoxicity and must be modified for satisfactory safety [85].

### Inorganic materials and combinatorial nVGDSs

Inorganic materials have an important role in nVGD and can be incorporated into hybrid materials to optimise nVGDSs, e.g. nanoporous silica-PEI nanoparticles [86]. For example, graphene binds to single-stranded DNA effectively but not to double-stranded DNA and can protect oligonucleotides from enzymatic cleavage. Graphene has recently been investigated for gene delivery applications, mostly using PEI-functionalised graphene oxide (GO) for the delivery of pDNA. Graphene and its derivatives can be modified and functionalised so that they do not exhibit acute or chronic toxicity, and can be cleared from the body over time. They can thus be used for biomedical applications including TE [87]. Li et al. used the EDC/sulpho-NHS crosslinking reaction to alter the dynamics of PAMAM-conjugated gold nanoparticles (AuPAMAM). The sMUA AuPAMAM constructs showed the highest stability, gene transfection efficacy, and a reasonable cytotoxicity profile [19].

Inorganic CaP coatings have been investigated to improve the vectors’ property. Mineral coatings resulted in widely variable transfection, and optimised coatings led to greater than tenfold increases in transgene expression by multiple target cell types when compared to standard techniques [88]. Layered double hydroxides (LDHs), commonly known as hydrotalcite-like materials and anionic clays, can be used as potential vectors because of their low cytotoxicity, good biocompatibility, and total protection of loaded DNA vaccines and LDH nanoparticles, which can be taken up by MDDCs efficiently and have an adjuvant activity for DC maturation [89, 90]. The novel vector SiO_2_@LDH, which is composed of core–shell nanoparticles with mesoporous silica as the core and LDH as the shell, activated macrophages and thereby enhanced systemic immune responses in animals, delivering HBVsAg DNA vaccine [91]. However, further in vivo experiments still need to be conducted to verify this kind of nVGV for future clinical application. Functional magnetic nanoparticles can also be used for gene delivery, either in the stand-alone form or as a modification of other chemical vectors; these have been reviewed by Xing et al. [92]. Cationic polymers and liposomes have their own advantages, and their combination contribute to the improvements in the property of hybrid nVGDS, e.g. lipopolyplexes [93]. Additionally, ultrasound and other physical methods have been used to modulate the transfection process for TRR [94, 95].

## Non-viral gene delivery systems for tissue repair and regeneration

**Fig. 2:**
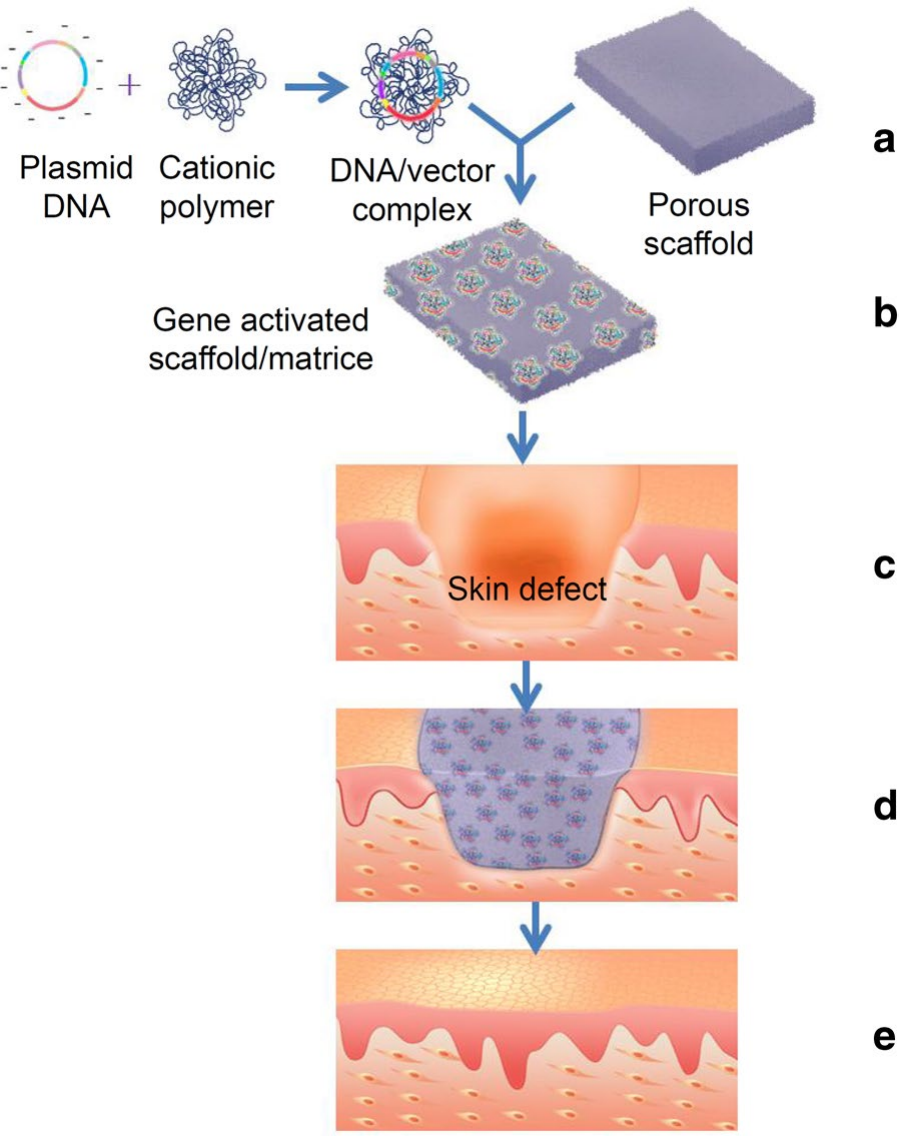
Schematic illustrating the construction of a gene activated scaffold/matrice and its application in skin defect. (a) Formation of a plasmid DNA/cationic polymer complex which is then loaded onto a scaffold. (b) A gene activated scaffold/matrice. (c) A deep skin defect. (d) Transplantation of a gene activated scaffold/matrice which fills the skin defect. (e) vascularization of the scaffold accompanied with repair and regeneration of the skin

Tissue repair and regeneration in critical defects is based on the induction, restoration and enhancement of the capability of self-repair or TE in different tissues [96]. The key requirements of an nVGDS for TRR are good biocompatibility and a high transfection rate [97, 98]. A typical model of non viral gene therapy for TRR is gene activated scaffold/matrice, such as polyplex loaded dermal scaffold (Fig. 2). However, different tissues have their unique characteristics that demand specific biomaterials, stimulators or inhibitors [99–103].

### Bone

Bone is the primary structural component of the body, characterised by its rigidity and hardness, and is a particularly active tissue, responsible for a wide range of functions [104]. Factors that regulate bone formation are usually involved in bone healing and engineering, e.g. bone morphogenetic proteins (BMP) [105] and platelet-derived growth factor (PDGF) (Table 2) [106].

**Table 2:** Typical examples of nVGDS for bone tissue repair and regeneration

Chemical vector	Scaffold/matrices	Wound type	Animal/cell	DNA/RNA	References
PEI	Collagen scaffold	Calvarial defects	Fisher 344 rats/human BMSCs	pDNA-PDGF-B	[109]
PEI coated with PEG	Poly-(ε-caprolactone) scaffold coated with poly-(d,l-lactide)	None	C2C12 cells	pDNA-BMP-2	[110]
PEI/FuGENE6	Calcium phosphate cement scaffold	Calvarial defects	Mice	pDNA-caALK6 and pDNA-Runx2	[111]
PEI	PEG hydrogels	None	hMSCs	siNoggin or miRNA-20a	[112]
Cationized gelatin microspheres (CGMS)	Oligo (poly(ethylene glycol) fumarate) (OPF) hydrogel	Cranial defects	Rat	pDNA-BMP-2	[114]
TAPP/gelatin microparticles (GMPs)	PPF scaffold	Cranial defect	Rat	pDNA-BMP-2	[115]
Chitosan-disulfide-conjugated low molecular weight-PEI	None	None	MG-63 osteoblast cells and stem cells	pDNA-BMP-2	[116]
(K)16GRGDSPC	PLGA-[ASP-PEG]n matrices	Segmental femoraldefects	Rabbit/rabbit-derived BMSCs	pDNA-TGF-b1	[118]
FuGENE6™	None	None	Rat/osteoblasts	pDNA-TGF-b1	[119]
Lipofectamine 2000	None	None	BMSC	AntimiR-138 (oligonucleotide)	[120]
Lipid (DOTAP-2-dioleoyl-sn-glycero-3-phosphatidylethanolamine/DOTAP-cholesterol)	None	None	Osteoblastic cell lines (MG63 and MC3T3-E1)	pDNA-β-gal	[121]
Calcium phosphate (CAP)	PLL film	None	Human osteoblasts	ShRNA(mouseSpp1andBglap-rs1)	[123]
Nanohydroxyapatite	Colagen-nHA scaffold	Cranial defect	Rat/MSCs	pDNA-VEGF and pDNA-BMP2	[126]
PEI-LA	Gelatin/collagen scaffolds	Subcutaneous implantation model	Rat	pDNA-bFGF and pDNA-BMP-2	[127]
Lipofectamine 2000 (coprecipitated within apatite)	PLGA films	None	C3H10T1/2 cell	pDNA-β-gal	[128]

Polymer-based delivery systems are favoured by many studies because polymers can be readily modified for various purposes. The most common synthesised cationic polymer vector is PEI and the branched form has shown significantly higher gene transfer efficiency than the linear form [107]. A molecular weight of 25 kDa has shown the highest transfection efficiency [108]. In the literature, Elangovan et al. [109] incorporated branched PEI (25 kDa)/pPDGF-B into collagen scaffolds as an attempt to optimise parameters of the PEI/DNA complex for the best balance between transfection efficiency and cytotoxicity. The PEI/DNA complex achieved a high transfection efficiency at the optimal N/P ratio of 10. Then, they applied the gene-activated scaffolds onto calvarial defects in Fisher 344 rats. The pPDGF-B-activated scaffold favoured cellular attachment and promoted cellular proliferation in vitro; it also promoted osteogenesis and demonstrated superior tissue regeneration efficacy in calvarial defects in rats when compared to the empty defect and empty scaffold groups. Reckhenrich et al. [110] used PEI (branched, 25 kDa)/pBMP-2 complexes, coated with an anionic PEG copolymer for enhanced stability, to activate a poly(d,l-lactide) coating of a poly(ε-caprolactone) scaffold (BMP-2-COPROGPDLLA-coated PCL scaffold) and applied it to initiate differentiation of relevant cells, e.g. myoblasts in vitro. With optimised gene doses, cells on the BMP-2-COPROGPDLLA-coated PCL scaffold secreted increased osteocalcin and osteopontin compared to the control group, suggesting a transdifferentiation of C2C12 cells into the osteoblastic phenotype with BMP-2-COPROGPDLLA-coated PCL scaffolds. This system has promise to enhance bone healing and the integrity of biodegradable implants. Another example is the sophisticated system consisting of dural plasmids, poly(ethyleneglycol) (PEG)-block-catiomer (PEG-b-P[Asp-(DET)]), and a CaP-cement scaffold. This system had good bio-compatibility. The plasmids encode osteogenic factors, activin receptor-like kinase 6 (caALK6), and runt-related transcription factor 2 (Runx2) [111]. Osteogenic differentiation was induced on mouse calvarial cells to a greater extent than when PEI or FuGENE6 were used [111]. Moreover, branched PEI (25 KDa)/siRNA or miRNA complexes were encapsulated within the PEG hydrogel, thereby delivering the nucleic acids in situ to guide stem cell osteogenic differentiation with sustained and localised RNA release [112].

Modified natural polymers have attracted much attention because of their better biocompatibility and lower cytotoxicity compared with synthesised polymers. Combining natural and synthesised polymers to optimise nVGDSs has been explored. Kasper et al. [113] developed a gene delivery system (GDS) consisting of gene vectors cationised with gelatine microspheres (CGMS) embedded within a crosslinked oligo(PEG fumarate) (OPF) hydrogel network. This system is a candidate material for the sustained, controlled release of pDNA. They examined the bone formation response to release of pBMP-2 from hydrogel composites in a critical-size rat cranial defect model after 30 days; the results showed lack of enhancement in bone formation, and the reason was considered as insufficient release of the DNA from the composite [114]. Chew et al. investigated the delivery of pBMP-2 in the form of polyplexes with a biodegradable branched triacrylate/amine polycationic polymer (TAPP) that were complexed with gelatine microparticles (GMPs) loaded within a porous TE scaffold. More specifically, the study investigated the interplay between TAPP degradation, gelatine degradation, pDNA release, and bone formation in a critical-size rat cranial defect model. The results showed that composite scaffolds containing GMPs complexed with TAPP/pDNA polyplexes did not result in enhanced bone formation, as analysed by microcomputed tomography and histology, in a critical-size rat cranial defect at 12 weeks postimplantation compared with those loaded with naked pDNA, but with slower release of pDNA than control groups. The results demonstrate that polycationic polymers with a slow degradation rate can prolong the release of pDNA from composite scaffolds and suggest that a GDS comprising biodegradable polycationic polymers could be designed to release pDNA in an intact polyplex form [115]. Zhao et al. delivered pBMP-2 via a delivery system of chitosan-disulphide-conjugated low molecular weight PEI (CS-ss-PEI), the transfection efficiency of which was significantly higher than that of PEI (25 kDa) and comparable to that of Lipofectamine. Inducing in vitro osteogenic differentiation, CS-ss-PEI4-mediated BMP-2 gene delivery showed a stronger effect in MG-63 osteoblast cells and stem cells in terms of alkaline phosphatase activity and mineralisation compared with PEI (25 kDa) and lipofectamine [116].

Peptide, as a component of nVGDS, usually enhances membrane activity and targeting ability. Shekaran et al. [117] conducted a study on a system of protease-degradable PEG synthetic hydrogel, functionalised with a triple helical, alpha2beta1 integrin-specific peptide (GFOGER). The hydrogel was applied to murine radial critical-sized defects and the results showed that this GFOGER hydrogel provided sustained in vivo release of encapsulated molecules, increased osteoprogenitor localisation in the defect site, enhanced bone formation, and induced defect bridging and mechanically robust healing at low BMP-2 doses, which stimulated almost no bone regeneration when delivered from collagen sponges. Though this system delivered the protein BMP-2, it has the potential to be used for gene delivery as well. In another study, transforming growth factor beta 1 (TGF-β1), a growth factor that regulates osteogenic differentiation of bone marrow stromal cells (BMSCs), was delivered by a novel nVV (K)16GRGDSPC that was chemically linked to bioactive bone matrices PLGA-[ASP-PEG]n. Applying these TGF-b1-activated matrices to 15-mm-long segmental rabbit bone defects significantly accelerated bone regeneration compared to control groups [118].

Traditional lipid-based delivery systems, e.g. Lipofactamine 2000 and FuGENE, have stable transfection efficiencies and are commercially available and, hence, are widely used in research. For example, Macdonald et al. adopted FuGENE6 to transfer the osteoinductive growth factor gene TGF-β1 to osteoblasts. The genetically modified osteoblasts showed greater levels of cellular proliferation when compared with addition of the same levels of recombinant TGFβ1, highlighting the advantages of delivering genetically modified cells over exogenous protein delivery for bone TE [119]. In another study, oligonucleotide antimiR-138 was delivered by Lipofectamine 2000-based formulations to BMSC to form stem cell sheets, which when applied to freeze-dried allograft bone (FDB) regenerated massive bone with good vascularisation [120]. The molecules 1,2-dioleoyl-3-trimethylammonium propane (DOTAP)-2-dioleoyl-sn-glycero-3-phosphatidylethanolamine and DOTAP-cholesterol were used in another case to form a liposome to deliver β-gal plasmid to osteoblastic cell lines (MG63 and MC3T3-E1) and the 294T line. Later inclusion of transferrin was conducted to increase the expression. The results revealed that this liposome had a higher transfection rate in osteoblastic cell lines than in the 294T line. It also demonstrated great dependency between the transfection activity and the lipid formulation, the charge ratios of the complexes, the applied DNA dose, and the cell type [121].

Inorganic materials such as CaPs are favoured in bone regeneration for their capacity to increase constructs’ stiffness and strength. Calcium phosphates possess numerous advantages, which include favourable biodegradability and biocompatibility properties, good solubility, lower toxicity than silica, quantum dots, carbon nanotubes, or magnetic particles, good binding affinity to DNA, good stability, efficient cellular uptake and resorbability, gradual release and escape from the endosomal network, and cytosolic transport and nuclear localisation of composite particles [122]. Consider RNA/CaP nanoparticles as an example. Zhang et al. developed a new type of coating based on polyelectrolyte multilayers containing sequentially adsorbed active shRNA CaP nanoparticles for locally defined and temporarily variable gene silencing and poly(l-lysine) (PLL) [123]. This system, when applied to human osteoblasts, presented efficient control of bone formation [123]. Hydroxyapatite (HA) is a member of the family of CaPs and is known as the mineral component of bone; it can be used as a component of an nVGDS [124]. Notably, self-assembling apatite hybrid materials have enabled the development of bi-/multi-molecular templates because natural bone is an outcome of a multi-molecular template co-assembly process [125]. With respect to gene therapy, the nanohydroxyapatite (nHA) vector delivered dural genes encoding for VEGF and BMP-2 to mesenchymal stromal cells (MSCs), and the stem cell/nHA-mediated gene delivery markedly enhanced tissue vascularisation and bone healing [126]. Other materials, e.g. alginate, can also deliver pDNA, e.g. pBMP-2, to MSCs and have promoted bone regeneration in a goat spinal cassette implantation model [105].

Hybrid delivery systems can be an attractive approach to improve the properties of nVGDSs. For example, lipid and polymer integrated materials, e.g. PEI modified with linoleic acid, were used to investigate the expression levels of basic fibroblast growth factor (bFGF) and bone morphogenetic protein-2 (BMP-2) in a rat subcutaneous implant model using different scaffold materials such as gelatine and collagen [127]. An organic/inorganic hybrid was developed by incorporating pDNA encoding for the β-gal gene complexed with Lipofectamine 2000 (DNA-Lipoplex) co-precipitated within apatite loaded onto PLGA films to integrate inductivity and conductivity [128]. The results showed that the coprecipitation of DNA-lipoplexes resulted in the highest transfection efficiency in all groups. It was believed that coprecipitation of the DNA-lipoplexes into biomimetically nucleated apatite resulted in better spatial distribution, higher stability, and higher transfection efficiency of DNA delivery [128].

### Skin

Skin is the largest organ in the human body. It functions as a natural barrier against many environmental hazards and maintains water balance between the outside and inside of the body. For this reason, skin regeneration is crucial for extensive burn or other skin injuries [129]. Fully constructed tissue-engineered skin is a major challenge because the structure of skin is stratified and the composition of skin is complex [130]. Genetically modified skin substitutes have been constructed to enhance skin disease treatment as reported in the literature [131]. Nucleic acids chosen for modification can be divided according to their encoding proteins or functions, e.g. growth factors, antimicrobial peptides, angiogenesis promoters, and scar formation inhibitors (Table 3) [131].

**Table 3:** Typical examples of nVGDS for skin tissue repair and regeneration

Chemical vector	Scaffold/matrices	Wound type	Animal/cell	DNA/RNA	References
TMC	Collagen–chitosan	Full-thickness burns	Porcine	pDNA-VEGF165	[132]
TMC	Collagen–chitosan/silicone membrane	Excisional skin defect	Porcine/fibroblasts	siRNA TGF-b1 pathway	[133]
PEI	PLA/PCL	Full-thickness skin defect	NIH-3T3 cells/C57BL/6J mice	pDNA-KGF	[134]
PEI	Collagen scaffold with a copoly-mer P6YE5C	Full-thickness skin defect	Nude mice/NIH-3T3	pVF1164-hVEGF^165^	[135]
PEI	PELA scaffold	Subcutaneous implantation	SD rats/HUVEC	pDNA-VEGF and pDNA-bFGF	[136]
PEG	Collagen scaffold	Full thickness skin defect	Rat/fibroblast	microRNA (miR)-29B	[137]
PLL-g-PEG polymers	Fibrin hydrogels	Full-thickness excisional skin defect	Healthy or diabetic rats/COS-7 cells	pDNA-HIF-1α	[138]
DMAEMA/PAA (PH responsible)	Polyurethane (PUR) scaffold	Nonhealing skin wounds	Diabetic rats/human cervical cancer cells	siRNA silencing GAPDH gene	[139]
Lipofectamine™ 2000	Collagen gels	Full-thickness burn	Rat/HaCaT cells	pDNA-EGF	[140]
None	None	Infected full-thickness burn	Human keratinocyte progenitor cell line (NIKS)	Plasmid-hCAP-18	[142]
None	None	Full-thickness burn with sepsis	NIKS human keratinocyte cell line	Plasmid-hBD-3/mice	[143]

Derivatives of natural cationic polymers are frequently in use in TE research, e.g. *N*,*N*,*N*-trimethyl chitosan chloride (TMC). Guo et al. [132] developed pDNA encoding VEGF-165-activated collagen–chitosan dermal equivalents to heal full-thickness burns in a porcine burn wound model via TMC, a derivative of chitosan. They demonstrated that the TMC/pDNA-VEGF group had a significantly higher number of newly-formed and mature blood vessels, and displayed the fastest regeneration of the dermis. Complete repair of the burn wounds with normal histology was observed after ultra-thin skin grafting was performed on the regenerated dermis 14 days later. Moreover, the tensile strength of the repaired tissue increased along with the time prolongation of post grafting, resulting in a value of approximately 70% of the normal skin at 105 days. Concerning the improvement of skin scar formation, gene-activated scaffolds have also been applied to advance skin-regeneration with inhibited scarring via an siRNA-loaded collagen–chitosan–silicone membrane bilayer dermal equivalent (BDE) [133]. To yield a bioactive RNAi-functionalised matrix for skin regeneration with inhibited scarring, the BDE was combined with TMC/siRNA complexes that could induce suppression of the TGF-b1 pathway. In a static 3D culture in vitro, the fibroblasts within the RNAi-BDE were able to internalise the siRNAs complexes and exhibited repressed TGF-b1 expression over 14 days. Stable gene-knockdown of TGFb1 for the wounds treated by RNAi-BDE was confirmed in a porcine excisional model in vivo. The expressions of Col I, Col III, and a-SMA were also down-regulated, indicating quantitative scar reduction. After the treatment of RNAi-BDE and ultra-skin grafting for 73 days, the RNAi-BDE induced a regenerated skin that was extremely similar to normal skin in terms of gross appearance and histological assessment [133].

The synthetic cationic polymer PEI, used in bone engineering, is also widely used in researches on skin regeneration. Engineered nanofibre PLA/PCL scaffolds, loaded with PEI/plasmids encoding keratinocyte growth factor (KGF) in a layer-by-layer manner to reach a desired ratio of PEI:DNA, provided highly efficient controlled DNA delivery and improved healing of full-thickness wounds in mice [134]. The PEI/plasmids encoding human vascular endothelial growth factor (VEGF) polyplexes were incorporated onto an Integra matrix. The gene-activated dermal scaffolds applied to nu/nu mice full-skin defects promoted skin construct vascularisation [135]. In another study, PEI was mixed with pDNA in solution, emulsified with PELA and PEG, then electrospun into fibres and lyophilised and multiple releases of polyplexes of VEGF and bFGF plasmids from electrospun fibrous scaffolds were achieved toward the regeneration of mature blood vessels in a subcutaneous wound animal model [136]. This study showed a low initial burst release followed by sustained release for about 4 weeks. The in vitro study demonstrated that the released pDNA from fibrous mats promoted cell attachment and viability, cell transfection and protein expression, and extracellular secretion of collagen IV and laminin. Furthermore, the pDNA polyplex-encapsulated fibres alleviated the inflammation reaction, enhanced the generation of microvessels, and improved the formation of mature vessels compared with pDNA polyplex-infiltrated fibrous mats. This clearly indicated the advantage of DNA encapsulated scaffolds [136].

Other cationic polymers used in skin TE research include PEG, PLL, 2-dimethylaminoethyl methacrylate (DMAEMA), and 2-propyl acrylic acid [137–139]. In a wound healing study, Monaghan et al. loaded miR-29b/PEG-based vector complexes into collagen scaffolds to examine their effects on ECM remodelling following cutaneous injury. They found reduced expression of collagen types I and III in fibroblast cultures, an effect that persisted for up to 2 weeks. These scaffolds were then tested in vivo in full thickness rat wounds. Compared with controls, the treated rats displayed reduced wound contraction, improved collagen type III/I ratios, and an increased ratio of MMP-8:TIMP-1 in a dose-dependent fashion [137]. Thiersch et al. described a system of transient gene expression by PLL-g-PEG polymer-mediated pDNA [encoding a truncated form of the therapeutic candidate gene hypoxia-inducible transcription factor 1alpha (HIF-1alpha)] delivery in vitro to induce angiogenesis. HIF-1alpha is the primarily oxygen-dependent regulated subunit of the heterodimeric transcription factor HIF-1, which controls angiogenesis among other physiological pathways. The HIF-1alpha gene delivery increased the number of endothelial cells and smooth muscle cells, precursors for mature blood vessels, during wound healing [138]. The study by Nelson et al. showed that nanoparticles, composed of the siRNA-silencing GAPDH gene and a pH-responsive smart polymer of DMAEMA and PAA, could be incorporated into injectable polyurethane scaffolds for the purpose of gene silencing in non-healing skin wounds [139].

Several lipid-based systems, e.g. Lipofectamine 2000, commercially available are often adopted in the routine use for skin regeneration research [140, 141]. HaCaT cells, an immortalised cutin cell line isolated from adult skin, were transfected with pDNA-EFG via Lipofectamine, providing seed cells with stable expression of EGF. The HaCaT-EGF cells then incorporated into skin substitutes and applied to a burn wound in an animal model demonstrated promoted wound healing. Such cells provide a useful experimental tool for the study of epidermal organisation, differentiation, and skin appendage regeneration.

Several kinds of commercial non-polymer non-lipid-based vectors have been applied in many studies [142, 143]. Using an nVV named the pUb-Bsd vector, Thomas-Virnig et al. genetically modified the novel, non-tumorigenic, pathogen-free human keratinocyte progenitor cell line (NIKS) to express the human cathelicidin HDP in a tissue-specific manner. The genetically modified bioengineered human NIKS skin tissue expressed elevated levels of cathelicidin, possessed key histological features of normal epidermis, and displayed enhanced antimicrobial activity against bacteria in vitro. Moreover, in an in vivo infected burn wound model, this tissue resulted in a two-log reduction in a clinical isolate of multidrug-resistant *Acinetobacter baumannii* [142]. Gibson et al. developed a bioengineered human skin tissue with enhanced expression of a host defence peptide, human β defensin-3 (hBD-3), to treat infected wounds and demonstrated improved healing of infected wounds [143].

Given the above developments in nVGDSs involved in skin regeneration, skin construct vascularisation, anti-inflammatory regulation, scar inhibition, and skin appendage regeneration are crucial aspects and should be taken into consideration when designing nVGDSs. The involvement of stem cells, e.g. hair-follicle stem cells, adipose-derived stem cells and mesenchymal stem cells, can promote skin repair and regeneration when properly induced [144].

### Cartilage

Cartilage is a non-vascular form of connective tissue with a supporting and protective function. It is composed of chondrocytes embedded in a matrix that includes chondroitin sulphate and various types of fibrillar collagen [145]. Unlike bone or skin, articular cartilage lacks the intrinsic ability to naturally regenerate because of its avascularity and lack of mobility of the chondrocytes that reside within the dense cartilaginous matrix [146].

Cationic polymers are an advantageous option for cartilage non-viral gene therapy (Table 4). Porous chitosan scaffolds with embedded hyaluronic acid/chitosan/pDNA nanoparticles encoding TGF-β1 induced DNA controlled release, transfected chondrocytes, and promoted cell proliferation [147]. PLGA nanoparticles were used to mediate SOX9 gene delivery in hMSCs and induce chondrogenesis [148]. Culturing rat bone marrow cells on blank PLGA scaffolds with PEI-complexed insulin-like growth factor-1 (IGF-1) resulted in growth and chondrogenic differentiation of these cells [149]. Babister et al. examined the potential of SOX-9 to transfect human bone marrow stromal cells and articular chondrocytes encapsulated within alginate polysaccharide microcapsules to promote chondrogenesis in vitro and in vivo. They confirmed that SOX-9 gene delivery enhanced chondrogenesis in targeted cell populations [150]. Primary chondrocytes were genetically modified with plasmid-encoding bone morphogenetic protein-7 (BMP-7) via the commercially available non-viral Turbofect vector, a proprietary cationic polymer. The genetically engineered cells were then implanted into gelatine-oxidised dextran scaffolds and cartilage tissue formation was investigated in auricular cartilage defects in vivo in New Zealand white rabbits over 4 months. There was a strong effect of exogenous BMP-7 on matrix synthesis and chondrocyte growth and significantly better cartilage healing with BMP-7-modified (transfected) cells than in the non-modified (non-transfected) group or the control [151].

**Table 4:** Typical examples of nVGDS for cartilage repair and regeneration

Chemical vector	Scaffold/matrices	Wound type	Animal/cell	DNA/RNA	References
Hyaluronic acid/chitosan	Chitosan scaffolds	None	Chondrocytes	pDNA-TGF-β1	[147]
PLGA nanoparticles	None	Subcutaneous implantation model	Female BALB/c mice/hMSCs	pDNA-SOX9	[148]
PEI	PLGA scaffolds	None	Rat/BMSCs	pDNA-IGF-1	[149]
Alginate polysaccharide microcapsules	None	None	hBMSCs and articular chondrocytes	pDNA-SOX9	[150]
Turbofect	Gelatin-oxidized dextran scaffolds	Auricular cartilage defect	New Zealand (NZ) white rabbits	pDNA-BMP7	[151]
FuGENE 6	PGA scaffold	Osteochondral defects in spatellar groove	Male Chinchilla bastard rabbits/chondrocytes	pCMVhIGF-I	[152]
GenePORTER™ 2 (GP2)	Collagen scaffolds	None	MSCs	Plasmid endostatin	[153]

Cartilage engineering using lipid-based delivery systems is less reported. Using the non-liposomal lipid formulation FuGENE 6, engineered cartilage with chondrocytes overexpressing a human IGF-I gene was constructed. The most enhanced articular cartilage repair and reduction of osteoarthritic changes in the cartilage occurred adjacent to the defect, and the enhancement of the repair of osteochondral defects was presented in a manner dependent on the duration of cultivation [152]. Another lipid-mediated transfection reagent GenePORTE 2 was used to deliver endostatin plasmid to MSCs via collagen scaffolds. The anti-angiogenic effect of overexpressed endostatin was believed to promote articular cartilage repair [153].

### Ligaments and tendons

Ligaments and tendons are complex composite materials. They are typically described as dense fibrous connective tissues that attach muscles to bones, and bones to bones, respectively, and they possess a high tensile strength that is crucial in mediating the normal movement and stability of joints [154].

Genes that can be delivered via nVGDSs to promote tendon healing include BMP-14, PDGF-B and fibromodulin [155–157] (Table 5). Suwalski et al. [155] used inorganic materials, i.e. MCM mesoporous silicas modified with amino or carboxyl groups, to encapsulate PDGF-B genes to transfect in vivo rat Achilles tendon, and accelerate Achilles tendon healing. Bolt et al. [156] delivered BMP-14 gene in a rat model of Achilles tendon injury to promote healing and increases tendon tensile strength. Delalande et al. [157] delivered fibromodulin gene via a liposomal-based system in a rat Achilles tendon injury model. Polylactic-co-glycolic acid nanospheres were prepared and incorporated with plasmids expressing enhanced green fluorescence protein and miRNA for inhibiting the transforming growth factor-b1 gene expression [158]. The results demonstrated that cultured tenocytes could be effectively transfected by means of nanosphere/plasmids. The expression of transforming growth factor-b1 was significantly downregulated in healing chicken flexor tendon treated with nanosphere/plasmids [158].

**Table 5:** Typical examples of nVGDS for tendon repair and regeneration

Chemical vector	Scaffold/matrices	Wound type	Animal/cell	DNA/RNA	References
Amino- and carboxyl-modified MCM-41 mesoporous silica nanoparticles (MSN)	None	Achilles tendon injury	Rat/primary tenocytes	pDNA-PDGF-B	[155]
Histidylated vectors (Lip100 and PTG1)	None	Achilles tendon injury	Wistar rats/tenocytes	pDNA-CEP4-FBM	[157]
Polylactic-co-glycolic acid nanospheres	None	Injured flexor tendon	Chicken	miRNAplasmid suppressing TGF-b1	[158]

Ligament repair involves a variety of factors such as BMP-7, TGF and PDGF, and cells including fibroblasts and myoblasts [159]. Multiple strategies have been developed to heal ligament injuries via gene therapy, mostly via virus-based delivery. Recent studies have highlighted the role of stem cells in ligament engineering and non-viral delivery methods [159]. Furthermore, lipid bubbles, created from 1,2-distearoylsn-glycero-3-phosphocholine and PEG 40 stearate, combined with insonation, were found to facilitate gene transfection of periodontal tissue when they were injected into the labial periodontal tissue [160].

### Other tissues

Applications of nVGDs and stem cells in TE have broadened with a deepening understanding of their biological behaviours. Repair and regeneration of solid organs have been reported in the literature [161, 162]. It has also been demonstrated that previously difficult transfecting cells, including neurocytes [163–165] and stem cells [166–170], are promising for gene transfer via various nVGDSs.

TE associated with nVGDS for solid organs has been reported for the repair and regeneration of cardiac and hepatic tissues (Table 6). Marsano et al. [161] used channelled elastomeric scaffolds delivering pVEGF in a mouse model of myocardial infarction, and the VEGF-expressing patches displayed significantly improved engraftment, survival, and differentiation of cardiomyocytes. Chien et al. used a polyurethane-grafted short-branch polyethylenimine copolymer via amphiphatic carboxymethyl-hexanoyl chitosan matrices to deliver microRNA122 (miR122) [162]. They suggested that the delivery system shortened the time of iPSC differentiation into hepatocytes. The miR122-iPSC-Heps may represent a feasible cell source and provide an efficient and alternative strategy for hepatic regeneration in acute hepatic failure (AHF).

**Table 6:** Typical examples of nVGDS for solid organ repair and regeneration

Chemical vector	Scaffold/matrices	Animal model	Cell	DNA/RNA	References
None	Channeled elastomeric scaffolds	Mouse model of myocardial infarction	Neonatal cardiomyocytes	pDNA-VEGF	[161]
Polyurethane-graft-short-branch polyethylenimine copolymer (PU-PEI)	Amphiphatic carboxymethyl-hexanoyl chitosan (CHC)	BALB/c nude mice model of acute hepatic failure	iPSCs	MicroRNA122 (miR122)	[162]

Neurons are suggested to have little capacity to regenerate. However, delivery of NA-encoding neuron growth factor is promising for promoting repair and regeneration of injured neuron tissues (Table 7). Spinal cord injury (SCI) models are often reported in the literature. Jeffery et al. [163] used magnetic nanoparticles (MNPs) via systemic delivery and found uptake of MNPs in areas of SCI associated with breakdown of the blood–brain barrier (BBB) within 6 h of injury, suggesting a therapeutic window of opportunity for systemic delivery of therapeutic NA. De Laporte et al. [164] delivered lipoplexes (Transfast) loaded on ECM-coated PLG 3D bridges in a rat spinal cord hemisection model. The transgene expression levels were two-fold greater than naked plasmid and the expression with lipoplexes persisted for at least 3 weeks. This system is suitable for neuron regeneration, which is a lengthy process, and the reporter genes in this delivery system can be replaced with therapeutic genes to further promote neuron tissue repair. The same research group adopted pGF and pNGF delivered by lipoplexes (Transfast) on poly(lactide-co-glycolide) (PLG) bridges that were surfaced with a layer of fibronectin [165]. This surface immobilization strategy enabled patterned gene delivery in vitro and in vivo.

**Table 7:** Typical examples of nVGDS for neuronal repair and regeneration

Chemical vector	Scaffold/matrices	Wound type	Animal/cell	DNA/RNA	References
Melittin-modified polymers	None	None	HeLa and neuron-like PC-12 cells	pDNA-GFP	[85]
Lipoplexes (Transfast)	ECM-coated PLG three-dimensional bridges	Spinal cord hemisection injury	Rat	pDNA-firefly luciferase and pDNA-b-galactosidase	[164]
Lipoplexes (Transfast)	Poly(lactide-co-glycolide) (PLG) bridges	Spinal cord hemisection injury	Rat/primary dorsal root ganglion (DRG) neurons and HEK293T cells	pDNA-GF pDNA-NGF	[165]

Stem cells are promising for the TRR of various tissue defects (Table 8). These hard-to-transfect cells are being investigated to achieve higher transfection efficiency using different nVGDSs [166, 167]. A PEI-based delivery system can reach an efficiency rate of 75% in transfecting stem cells, and 3D scaffolds with nHA particles can affect 88.4% of the cells at day 7 [168]. Hydrogels constitute a recent hot topic for gene delivery, especially in cases involving stem cells. Tokatlian et al. [169, 170] used a caged nanoparticle encapsulation (CnE) technique to deliver reporter genes into mMSCs via hyaluronic acid hydrogels, resulting in sustained release and minor cytotoxicity of the DNA polyplex that was in high dose in the hydrogel.

**Table 8:** Typical examples of nVGDS for stem cell based tissue repair and regeneration

Chemical vector	Scaffold/matrices	Cell	DNA/RNA	References
Polyethylenimine (PEI)	None	hMSCs	pDNA-green fluorescent protein (GFP)	[166]
Nanohydroxyapatite (nHA) particles	Collagen-nanohydroxyapatite	hMSCs	Reporter miRNAs (nanomiRs)	[168]
Spermine-introduced pullulan (spermine–pullulan)	Three-dimensional scaffolds of gelatin and beta-tricalcium phosphate (beta-TCP)	Rat MSCs	pDNA-Luciferase	[167]
Caged nanoparticle encapsulation (CnE) technique	Hyaluronic acid hydrogels	mMSCs	Reporter gene	[169]

### Major challenges and prospects for future development

Tissue repair and regeneration in a certain tissue is unique to some extent due to the different characteristics of the injured cells and extracellular matrix. The choice of a proper nVGDS for TRR should be made according to the property of the injured tissue and its potential regeneration pattern. Some of the most advanced methods for nVGD-mediated TE include the delivery of micro- or si-RNA [171–173], genetically engineered stem cell-based therapy [174–179], development of gene-activated scaffold platforms [180, 181], better spatiotemporal regulation in generating GAMs [182, 183], and more sophisticated nVGDSs [184]. For future clinical applications, economic and ethical considerations as well as the ease of use should be considered. Based on these concerns, nVGDSs for TRR are faced with the following challenges.

First, the mechanism of an nVGD is still unclear. The optimal balance between transfection rate and cytotoxicity in designing novel nVVs has not yet been achieved. How to exert sufficient therapeutic effects and minimise side effects when applying an nVGDS in TRR remains a major challenge.

Second, potential seed cells, such as stem cells, have been identified for TRR. However, they are difficult to successfully transfect. In vitro culture of these cells is lengthy and cost-inefficient, and in vivo culture is faced with even more difficulties. Ethical problems are also a major concern when cells are incorporated in spite of numerous ongoing clinical trials involving stem cells.

Third, choosing proper genes or combinations of genes is a major undertaking. It is generally associated with developing a better understanding of the physio-pathological role of different genes in TRR. Some known genes have exhibited excellent effects in promoting TRR, such as gene targeting FGF2, PDGF and VEGF for angiogenesis. However, the process of TRR is sophisticated and involves various genes and other biomolecules, and better combinations of genes are expected.

Given the massive amount of scientific evidence described above, it is anticipated that significant efforts will be made to tackle the key problem of the transfection efficiency/side effects balance. Additionally further investigations into the growth and management of seed cells in TRR, and the most suitable composition of multiple kinds of DNA, RNA and protein delivery for TE are expected.

## Conclusions

nVGDSs have been widely investigated and are promising for TRR. Though the detailed mechanisms underlying nVGDs remain unknown, barriers that impede efficient transfection via nVGDSs should be taken into account when designing new nVGVSs or platforms. Investigations into the delivery of interfering RNA or the combination of nucleic acids, optimised stem-cell management, and the incorporation of a advanced strategy such as three dimentional printing are promising trends in nVGDS-mediated TE. Further steps from bench-to-bed of the research results can be anticipated in the forthcoming decades.

## Abbreviations

TE: tissue engineering
TRR: tissue repair and regeneration
nVGDS: non-viral gene delivery systems
NAs: nucleic acids
pDNAs: plasmid deoxyribonucleic acids
siRNA: small interfering ribonucleic acids
miRNA: microRNAs
NA: nucleic acid
RNAi: RNA interference
VVs: viral vectors
nVVs: non-viral vectors
PEI: polyethylenimine
CaPs: calcium phosphates
VGCs: vector-gene complexes
GAMs: gene-activated matrices
nVGCs: non-viral vector/gene complexes
nVGD: non-viral gene delivery
CME: clathrin-mediated endocytosis
CvME: caveolae-mediated endocytosis
NPC: nuclear pore complex
NLS: nuclear location sequence
3D: three-dimensional
CPPs: cell-penetrating proteins
GAGs: glycosaminoglycans
PAMAM: polyamidoamine
DTS: DNA nuclear targeting sequence
NF: nuclear factor
LDHs: layered double hydroxides
BMP: bone morphogenetic proteins
PDGF: platelet-derived growth factor
GDS: gene delivery system
TGF-β1: transforming growth factor beta 1
BMSCs: bone marrow stromal cells
FDB: freeze-dried allograft bone
PLL: poly(l-lysine)
HA: hydroxyapatite
nHA: nanohydroxyapatite
hMSCs: human mesenchymal stromal cells
VEGF: vascular endothelial growth factor
TMC: *N*,*N*,*N*-trimethyl chitosan chloride
BDE: bilayer dermal equivalent
HIF-1alpha: hypoxia-inducible transcription factor 1alpha
IGF-1: insulin-like growth factor-1
